# Within but without: human rights and access to HIV prevention and treatment for internal migrants

**DOI:** 10.1186/1744-8603-5-17

**Published:** 2009-11-19

**Authors:** Katherine Wiltenburg Todrys, Joseph J Amon

**Affiliations:** 1 Health and Human Rights Division, Human Rights Watch, London, UK; 2 Health and Human Rights Division, Human Rights Watch, NY, NY, USA

## Abstract

Worldwide, far more people migrate within than across borders, and although internal migrants do not risk a loss of citizenship, they frequently confront significant social, financial and health consequences, as well as a loss of rights. The recent global financial crisis has exacerbated the vulnerability internal migrants face in realizing their rights to health care generally and to antiretroviral therapy in particular. For example, in countries such as China and Russia, internal migrants who lack official residence status are often ineligible to receive public health services and may be increasingly unable to afford private care. In India, internal migrants face substantial logistical, cultural and linguistic barriers to HIV prevention and care, and have difficulty accessing treatment when returning to poorly served rural areas. Resulting interruptions in HIV services may lead to a wide range of negative consequences, including: individual vulnerability to infection and risk of death; an undermining of state efforts to curb the HIV epidemic and provide universal access to treatment; and the emergence of drug-resistant disease strains. International human rights law guarantees individuals lawfully within a territory the right to free movement within the borders of that state. This guarantee, combined with the right to the highest attainable standard of health set out in international human rights treaties, and the fundamental principle of non-discrimination, creates a duty on states to provide a core minimum of health care services to internal migrants on a non-discriminatory basis. Targeted HIV prevention programs and the elimination of restrictive residence-based eligibility criteria for access to health services are necessary to ensure that internal migrants are able to realize their equal rights to HIV prevention and treatment.

## Introduction

Worldwide, far more people migrate within their country than out of it [1]. Internal migrants--as opposed to international migrants--are those individuals who change residence from one civil division to another within their country of origin. Reasons for migration are varied, but typically stem from social, political, or financial causes, or natural disaster. Urbanization and increased manufacturing in East and Southeast Asia have led to circular rural-urban migration in unprecedented numbers in Indonesia, Vietnam, and Cambodia, and to increased rural-rural and rural-urban migration in India [1]. In some cases, the lifting of restrictions on movement--as in South Africa in the post-apartheid era--have led to increased internal migration [1], and migration within countries in Eastern Europe and the Commonwealth of Independent States since the fall of the Soviet Union has been significant [2]. Intra-metropolitan migration has become increasingly common in Latin America as well [1].

The global financial crisis has seriously affected spending on HIV/AIDS services, and a March 2009 survey by the World Bank, the Joint United Nations Programme on HIV/AIDS (UNAIDS), and the World Health Organization (WHO) found that some countries are already facing drug shortages and other disruptions in HIV/AIDS treatment [3]. The report predicted that the crisis would further impact prevention and treatment programs, leading to increased illness, death, and the development of drug resistance. The financial crisis has particularly thrown into relief the plight of internal migrants as it has exacerbated health and social inequalities [3,4]. In declining markets, migrant workers are often the first to lose their jobs: By February 2009 in China, approximately 20 million migrant workers had been laid off or were unable to find work [5]. With fragile social support networks, the health-related consequences of unemployment for this population may be dire; returning to often rural and impoverished origins or seeking work in new locations may be equally difficult.

International human rights law guarantees individuals lawfully within a territory the right to free movement within the borders of that state [6], a commitment legally binding on all parties to the International Covenant on Civil and Political Rights (ICCPR) [7]. The Human Rights Committee, the ICCPR's monitoring body, has noted that liberty of movement is an "indispensable condition for the free development of a person." [8] The International Convention on the Elimination of All Forms of Racial Discrimination also supports the right to freedom of movement within a state [9]. But while such freedom of movement is assured by international law, it is not always respected in practice by states, as countries put restrictions on movement and limit services available to unofficial internal migrants.

Already marginalized and subject to stigma as a result of their migration status [10], migrants with HIV/AIDS are doubly stigmatized and are subject to neglect and exploitation [11]. Gaps in internal migrants' access to HIV/AIDS services--either as a result of official restrictions or logistical, cultural and linguistic barriers--have significant consequences: individuals are less able to access care and are increasingly vulnerable to infection and death, states are less able to realize the goals of universal access to treatment and reduction of the AIDS epidemic, and the public health community may face the emergence of drug-resistant strains resulting from interruptions in treatment [12]. This article describes some of the barriers to access to HIV/AIDS-related services faced by internal migrants when they move from their place of origin, highlighting three countries--China, Russia, and India--that have internal migration restrictions, and logistical, linguistic and cultural barriers to HIV/AIDS prevention and treatment. To successfully achieve global goals for reducing the burden of HIV and providing universal access to prevention and care, states must recognize the rights of internal migrants and their own obligations to eliminate barriers to care.

## Barriers to HIV/AIDS prevention and treatment facing internal migrants: China, Russia, and India

### The People's Republic of China

As a result of economic reforms, a surplus of rural labor and desperate rural poverty, internal migration has drastically increased in China in recent years. As of December 31, 2008, 140.4 million internal migrants in China worked outside their home village or township [13], an increase from only two million internal migrant workers two decades earlier [14]. Internal migrants make up a sizeable percentage of the urban population and workforce [15].

Through the system of *hukou*, the People's Republic of China requires the registration of every Chinese resident with the local authorities. Although the Chinese government has announced plans for its elimination [16], *hukou *allows individuals to live and work only where they are officially permitted [15], with one place of permanent *hukou *registration. *Hukou *status is inherited, so that children of rural-to-urban migrants are, like their parents, not registered urban residents [17]. Procedures to obtain temporary residence can be time-consuming, expensive, and difficult [18]. Only an estimated 40% of China's internal migrants typically obtain temporary or permanent permits [14].

While urban permit-holding residents in China have long been entitled to state-sponsored social welfare benefits including retirement pensions, food, education, and medical care, internal migrants still registered in their rural household of origin are denied such benefits [19]. Individuals without *hukou *are unable to access basic public services such as education [20] and health care [21], and therefore are forced to pay all costs [15,21]. Amnesty International has noted that the vast majority of internal migrants in China cannot afford insurance schemes and rarely visit doctors or hospitals [18]. Human Rights Watch has documented widespread lack of insurance coverage for migrant construction workers, despite government guarantees of medical and accident insurance [19]. Furthermore, lack of health care coverage for sick migrants has, in the past, been compounded by additional, harsh consequences: For example, internal migrant workers have been returned to their home province under armed guard after being found to be HIV positive [22]. Though China announced the abolition of such "custody and repatriation" in 2003 [23], recent reports suggest that similar practices of detention and removal purportedly for health reasons are still practiced, particularly during periods of heightened political concern [24].

A range of studies have documented the disproportionately high prevalence of HIV among internal migrants: Multi-city HIV surveillance data between 1995 and 2000 revealed that over two-thirds of the HIV cases were found among rural-to-urban migrants. In 2000, 85.4% of Beijing's and 74.4% of Shanghai's new HIV infections occurred among migrants [25,26]. Despite such high prevalence, and nationwide prevention campaigns in recent years, as well as studies calling urgently for HIV prevention programs addressing the particular circumstances of migrants [27], internal migrants in China have disproportionately low access to HIV/AIDS-related information [18,26,28]. United Nations reports have also remarked on the special vulnerability and difficulty of reaching with prevention programs the children of migrants, who lack access to the formal Chinese schooling system [29].

HIV-positive internal migrants' access to treatment remains extremely limited, confounded in part by the effects of the *hukou *system. Prior to 2003, ART was only available to the wealthy elite, as hospitals and clinics passed along to all patients the cost of HIV/AIDS examinations, tests, hospitalizations, treatment for opportunistic infections and ART treatment [22]. In 2003, the Chinese government announced a national HIV/AIDS treatment program--free to rural residents and poor urban residents--funded by national and provincial authorities [30]. However, despite such broad policy statements, universal HIV/AIDS treatment is far from a reality among the general population: In 2007, UNAIDS estimated that 190,000 people living with HIV were unable to access urgently needed ART in China, representing 81% of those in need [31]. Even when free treatment is ostensibly offered, delays in diagnosis and referral can create significant costs for the patient prior to the availability of free treatment, thus particularly disadvantaging migrants, who are not entitled to free basic health care [32].

The negative health consequences of the restrictive *hukou *system and related gaps in HIV/AIDS prevention and treatment for internal migrants have been exacerbated by the recent crisis in the world financial markets. For example, the loss of jobs in the export manufacturing sector, such as in the Pearl River Delta region, is anticipated to increase the number of migrant women working in the sex industry [33]. As unemployed internal migrants return to rural areas there is a potential for increased HIV transmission, as well as a risk that inadequate and weakened rural health systems will become overburdened [30]. Recognizing the current disparity in health care access, and widespread dissatisfaction, the Chinese government has recently announced plans for significant investment in basic health care services [35].

### The Russian Federation

Vestiges of an internal registration system also plague access to health care for internal migrants in Russia. In the former Soviet Union, *propiska*--a residence permit stamp on internal Soviet passports--strictly limited movement and residence. Although *propiska *was officially abolished by the federal government in the 1990s, local and regional governments retain restrictive systems of registration for both temporary visitors and residents [36]. While reliable statistics are unavailable, government officials have estimated that over a million unregistered individuals may live in Moscow alone [37].

In recent years, legislative and other changes have led to the simplification and relaxation of some registration requirements [36-38]. Federal law and policy provide for freedom of movement and, while requiring registration [39], envision it as a non-discretionary, notice-based system open to all. However, in practice, registration is cumbersome and expensive, and lack of registration status may have serious official or unofficial consequences for internal migrants. Instances of unregistered migrants unable to legally marry, vote, send their children to school, and receive public assistance, have all been reported [36]. Indeed, individuals who are legally in the country but lack local registration have also reportedly faced such harsh consequences as detention, police abuses or deportation [36,40,41].

While the Russian government is constitutionally required to provide free medical care to all citizens [42,43], regional authorities, responsible for the organization and financing of medical programs in their territories, regulate the conditions for access to medical care. Federally funded HIV treatment is officially provided free of charge to citizens [44,45], but in practice major challenges exist in access to free health care generally as a result of inadequate federal and regional funding [46]. UNAIDS estimated in 2007 that 159,000 individuals needing ART were not receiving it, as only 16% of those requiring ART had access to treatment [47]. Internal migrants especially face barriers, as registration is a precondition for entitlement to many free health services [48,49].

Human Rights Watch research has documented that internal migrants without registration are often denied both short-term (for purposes of Prevention of Mother to Child Transmission) and long-term antiretroviral treatment [50]. In Moscow, individuals must produce temporary registration and an official certificate of HIV-status in order to obtain ART at the Moscow AIDS Center. While unregistered international migrants may, in some cases, receive antiretrovirals for free, a non-resident requiring antiretrovirals will typically be directed to his or her city of origin to receive the treatment. Despite these barriers to accessing care, currently applicable Russian federal law on HIV/AIDS does not specifically address the particular challenges involved in providing HIV prevention, care and treatment services to migrants [44].

There is some preliminary indication that the global financial crisis may in fact lead to an increased movement into Russian cities, where the remaining registration systems prevent internal migrants from accessing some social services: According to the head of the Moscow Directorate of Internal Affairs, increasing unemployment as a result of the global financial crisis in Russia has lead to an influx of migrants from regions surrounding Moscow into the city in search of work. In addition to facing the restrictions detailed above, these internal migrants have been blamed for an increase in crime [51], and have encountered significant hostility and attacks [52].

### Republic of India

India, like China and Russia, has high rates of internal migration--both rural-rural and increasingly rural-urban [1]--complicated by diverse cultural and linguistic traditions. An estimated 258 million adults in India are migrants [53]. While poverty and internal mobility itself does not lead to HIV transmission, unsafe sex and a change in sexual networks may [54,55]. The World Bank has characterized migration and mobility, particularly for work purposes, as one of the major risk factors for HIV in India [56]. The national government's response to HIV/AIDS has recognized the key role that migrants have played in the on-going epidemic [57]. While the correlation between migration status and HIV infection in India may have been weakening in recent years [55], rising unemployment as a result of the financial crisis and the existence of return migration may have the potential to increase transmission [58].

Approximately 2.4 million people were living with HIV/AIDS in India in 2008 [59]. HIV prevention is seriously hindered by the low awareness of the disease among internal migrants, particularly from rural areas [56,57]. UNAIDS India representatives have called for awareness campaigns specifically targeting the sending areas for internal migrants [54], however HIV prevention activities can be hindered by the mobile nature of this population [60], language, and cultural barriers [53].

Significant HIV/AIDS treatment gaps exist for all groups throughout the country, but migrants also face particular challenges in accessing health care [59,61]. Health care is administered on a state-by-state basis in India, and in some states significant uncertainty exists among government officials as to whether state authorities are responsible for social welfare services to temporarily resident workers and their families [62]. Furthermore, internal migrants are often unable to use the government-issued "ration cards" outside their local home authority in order to access social services [63], and migrants may face significant logistical challenges and delays in procuring a new ration card [64]. Absent a ration card, it can be difficult to access even programs designed to provide health care to the poor, as some such services specifically target ration card holders [65]. Indeed, some local authorities reportedly refuse to provide ART entirely to individuals without ration cards [66]. In one area with extensive seasonal out-migration, a study concluded that internal migrants reported poorer health-seeking behavior than their non-migrant counterparts, a difference attributed to ignorance of behavioral risk factors, lack of knowledge of health facilities, and cultural and linguistic barriers [55].

Though not as severe as in some countries worldwide, the current global financial crisis has slowed economic growth in India and threatened to exacerbate preexisting levels of internal inequality [67]. Internal migrants are particularly vulnerable to increased unemployment and poverty, and the process of reverse migration has already begun [68]. The Governor of the Reserve Bank of India noted in February 2009 that social safety net programs in rural areas could help to mitigate the impact of the crisis for migrant workers who return home [69]; however, ART coverage throughout the country is plagued by broad gaps and failures and interruptions in treatment must be expected.

## International law

International human rights law guarantees individuals lawfully within a territory the right to free movement within the borders of that state [6], a commitment legally binding on all parties to the International Covenant on Civil and Political Rights [7]. International law also provides for the basic right to the highest attainable standard of health. This right, along with the principle of non-discrimination, implies a clear right to access a core minimum set of health services for migrants who move within their own state, including ART, without discrimination on the basis of social origin.

### Right to highest attainable standard of health

All individuals have the right to enjoy the highest attainable standard of health, a right which has been enshrined in international and regional treaties. According to the Universal Declaration of Human Rights (UDHR), " [e]veryone has the right to a standard of living adequate for the health and well-being of himself and of his family, including food, clothing, housing and medical care and necessary social services." [6] The International Covenant on Economic, Social and Cultural Rights also guarantees the right of everyone to the highest attainable standard of health, and requires states parties to take steps individually and through international cooperation to progressively realize this right via the prevention, treatment, and control of epidemic diseases and the creation of conditions to assure medical service and attention to all [70]. "Progressive realization" demands of states parties a "specific and continuing obligation to move as expeditiously and effectively as possible towards the full realization of [the right]." [71] According to the WHO, " [w]hen considering the level of implementation of this right in a particular State, the availability of resources at that time and the development context are taken into account. Nonetheless, no State can justify a failure to respect its obligations because of a lack of resources." [72] The concept of available resources is intended to include available assistance from the international community [73].

The right to health is further guaranteed by a number of other international human rights treaties and commitments. The Convention on the Rights of the Child binds states to "recognize the right of the child to the enjoyment of the highest attainable standard of health and to facilities for the treatment of illness and rehabilitation of health. States Parties shall strive to ensure that no child is deprived of his or her right of access to such health care services." [74] The right to health is also protected under the International Convention on the Elimination of All Forms of Racial Discrimination [9], the Convention on the Elimination of All Forms of Discrimination Against Women [75], the International Convention on the Protection of the Rights of All Migrant Workers and Members of Their Families [76], and the Convention on the Rights of Persons with Disabilities [77]. Additionally, governments committed in the 2001 Declaration of Commitment on HIV/AIDS to "promote and protect all human rights and fundamental freedoms, including the right to the highest attainable standard of physical and mental health" and "in an urgent manner make every effort to: provide progressively and in a sustainable manner, the highest attainable standard of treatment for HIV/AIDS, including the prevention and treatment of opportunistic infections, and effective use of quality-controlled anti-retroviral therapy in a careful and monitored manner to improve adherence and effectiveness and reduce the risk of developing resistance" [78].

To be consistent with the right to health, the health resources provided should have the characteristics of respect for medical ethics, cultural appropriateness, and respect for confidentiality. Indeed, " [a]ll health facilities, goods and services must be... respectful of the culture of individuals, minorities, peoples and communities, sensitive to gender and life-cycle requirements, as well as being designed to respect confidentiality and improve the health status of those concerned" [71].

### Principles of equality and non-discrimination

International law also establishes the fundamental principles of non-discrimination and equality. The Universal Declaration of Human Rights proclaims that " [e]veryone is entitled to all the rights and freedoms set forth in this Declaration, without distinction of any kind, such as race, colour, sex, language, religion, political or other opinion, national or social origin, property, birth or other status". [6] Additionally, under that Declaration, " [a]ll are equal before the law and are entitled without any discrimination to equal protection of the law" [6]. The ICCPR echoes the UDHR's proclamations against discrimination, binding states party to recognize the rights it guarantees without distinction of any kind, including based on race, colour, sex, language, religion, political or other opinion, national or social origin, property, birth or other status [7]. The ICCPR also notes the equality of all persons before the law and requires that the law prohibit discrimination and guarantee equal protection against discrimination on any ground, including the above-noted ones [7]. The Human Rights Committee, the ICCPR's monitoring body, has determined non-discrimination, equality before the law, and equal protection, to be basic principles in the protection of human rights [79]. Indeed, the Human Rights Committee, the ICCPR's monitoring body, has noted that states must eliminate all discrimination and indeed in some cases may need to take affirmative steps to realize the value of that guarantee [79].

### Non-discrimination in health

Numerous international and regional bodies have, considering the abovementioned right to the highest attainable standard of health and principle of non-discrimination, addressed specifically the prohibition on discrimination in health services. According to the Economic, Social and Cultural Rights Committee, the Covenant on Economic, Social and Cultural Rights' monitoring body, States must guarantee certain core obligations as part of the right to health, including ensuring non-discriminatory access to health facilities, particularly for vulnerable or marginalized groups; providing essential drugs; ensuring equitable distribution of all health facilities, goods and services; adopting and implementing a national public health strategy and plan of action with clear benchmarks and deadlines; and taking measures to prevent, treat and control epidemic and endemic diseases [71]. While the Committee notes the progressive nature of the right to health, it also points to the fact that states must immediately take steps to realize the right to health, and must immediately guarantee the exercise of the right without discrimination of any kind [71].

The right to health is thus centrally linked to the right to non-discrimination. Indeed, the Committee has noted that "the Covenant proscribes any discrimination in access to health care and underlying determinants of health, as well as to means and entitlements for their procurement, on the grounds of race, colour, sex, language, religion, political or other opinion, national or social origin, property, birth, physical or mental disability, health status (including HIV/AIDS), sexual orientation and civil, political, social or other status, which has the intention or effect of nullifying or impairing the equal enjoyment or exercise of the right to health...With respect to the right to health, equality of access to health care and health services has to be emphasized. States have a special obligation to provide those who do not have sufficient means with the necessary health insurance and health-care facilities, and to prevent any discrimination on internationally prohibited grounds in the provision of health care and health services, especially with respect to the core obligations of the right to health...." [71].

Discrimination against internal migrants--who are in fact citizens of the state in question--is banned under the Committee's Comments, which explicitly state that the Covenant prohibits discrimination based on "social origin." The ban against discrimination receives further confirmation when the Committee stresses each state's obligation to make health facilities and services accessible to everyone within the state's jurisdiction without discrimination, particularly the most vulnerable, so that health facilities, goods and services are within safe physical reach of "all sections of the population," "especially vulnerable or marginalized groups, such as ethnic minorities and indigenous populations, women, children, adolescents, older persons, persons with disabilities and persons with HIV/AIDS" [71]. Thus, the Committee findings make clear that the Covenant prohibits discrimination against internal migrants in receiving health care, and are an immediate call on all states parties to eliminate discrimination.

The Committee on the Rights of the Child has spoken specifically to the relationship between HIV/AIDS and the rights outlined in that Convention, determining that the right to non-discrimination should be one of "the guiding themes in the consideration of HIV/AIDS at all levels of prevention, treatment, care and support" [80].

## Discussion

In the history of the response to HIV/AIDS, governments have frequently sought to blame culturally different "others"-first, foreigners, and second, minorities, migrants and individuals considered socially "deviant" [81,82]. Internal migrants are often included in more than one of these categories, and have long struggled to gain access to HIV prevention information and treatment. As HIV programs seek to scale-up services and fulfill commitments to provide "universal access" to prevention and care, it continues to be controversial to include migrants among those who are entitled to care [83], and in some cases migrants are subject to treatment including deportation as a result of their very HIV status [84,85]. As with international migrants, whose rights are frequently denied, internal migrants' rights are often unrecognized [1,18,86].

China, Russia and India, like many countries worldwide, are rapidly scaling up provision of ART. Between 2004 and 2007, the estimated number of people receiving ART in China rose from 9,000 to 35,000 [31]. In Russia, the estimated number of people receiving ART rose from 3,000 in 2004 to 31,000 in 2007 [47]. In India, the estimated number of people receiving antiretroviral therapy increased from 28,000 in 2004 to 158,000 in 2007 [59,87]. But without the implementation of free treatment, the elimination of eligibility restrictions for access to care, an end to restrictions on internal migrants, and targeted programs to facilitate access to HIV prevention info and treatment, universal access goals will fail and internal migrants will continue to face barriers to accessing care.

First, states need to implement free ART for internal migrants on the same terms as local residents. Research has found that user fees constitute the main barrier to ART adherence, and that free care at point of service leads to improved uptake of HIV-related services, especially among the poorest users [88-93]. Lack of access to treatment from government-sponsored health sources also serves to push internal migrants toward self-medication or illegal clinics [94]. Such clinics and self-medication expose internal migrants to a host of health risks, including from counterfeit pharmaceuticals and unproven AIDS 'cures' [95].

States must also work to alleviate the hidden costs of receiving treatment. Research has shown significant additional costs to receiving treatment even for those people entitled to free ART: In India, free ART at government-run centers is complicated by transport costs which may include overnight stays near the clinic (especially given few centers in rural areas), private clinic fees paid after negative experiences with government clinics, the cost of vitamins and nutritious food, lost time waiting in government hospitals, payment for drugs at times of government stock outs, and costs for second-line drugs for individuals who developed resistance to first-line drugs [96].

Second, in countries that place formal or informal eligibility restrictions on access to health care, such restrictions based on social origin within different regions of the country need to be immediately eliminated. As noted above, the Economic, Social and Cultural Rights Committee directs that states have an immediate obligation to eliminate discrimination in health care provision, including discrimination based on "social origin." The obligation to ensure HIV/AIDS prevention and treatment to all individuals without discrimination is all the more acute, as antiretroviral medicines used in the prevention and treatment of HIV/AIDS are included as essential medicines in the core minimum of health care services nations have an obligation to provide [71,97]. Some sources, including the UN Special Rapporteur on the Right of Everyone to the Enjoyment of the Highest Attainable Standard of Physical and Mental Health, have argued that essential medicines, as part of the core of the right to health, are subject to immediate realization for the entire population rather than progressive [98].

Third, national governments need to remove restrictions on movement that prevent or delay internal migrants from establishing residence in urban areas. The harsh consequences and rights violations of restrictions on internal migration in some countries can include detention or deportation. Fear of such consequences may lead internal migrants to avoid HIV-related services even when they are available. Human Rights Watch has documented the chilling effect that fear of detention and deportation of foreign-born mothers can have on their Chinese partners' decisions to obtain *hukou *for their children and enroll them in school [20]. Human Rights Watch has also noted Chinese internal migrants' fear of contact with the official government services out of concern that they will be ejected from their city of residence [19]. In Russia, Human Rights Watch found that " [m]igrants with irregular status are more vulnerable to abuses and less willing to seek assistance from government agencies out of real fears that approaching any official person or body will result in a fine or expulsion" [99].

Finally, creating programs tailored specifically to internal migrants' needs is essential to uptake even of free HIV prevention and treatment services. The experience of free tuberculosis (TB) treatment programs is illustrative both as a model for other health services and in suggesting what targeted programs may be necessary to make even free care truly accessible to internal migrant populations. In many countries, TB treatment is widely provided free of charge by national governments to all individuals regardless of citizenship or residency status [100]. Provision of TB treatment is often more widely available within countries than HIV treatment--in India, for example, in 2006, 634 (100% coverage) Ministry of Health facilities in the country were providing Directly Observed Treatment, Short-course (DOTS) services for TB treatment [101], whereas in 2007, only 137 sites nationwide were providing ART [59]. Free universal TB treatment can serve as a model for the expansion of free HIV treatment, and existing TB services represent an opportunity for expanding access to HIV prevention, treatment, care and support, particularly in the context of HIV/TB co-infection.

Yet TB treatment for migrants is also a cautionary tale of the barriers that still exist when ostensibly free care is implemented without programs targeted to alleviate internal migrants' particular circumstances. In China, a country with one of the highest TB burdens in the world, the government has worked since at least 1978, and increasingly since 1991 with the initiation of the National TB Control Program, to implement the DOTS program, to increase TB treatment. In 2005 China had established TB coverage over 100% of the country (though quality concerns remained) [102]. However, migrant status remains a main reason for delays in diagnosis [103]. Indeed, without *hukou*, migrant workers rarely have access to free TB diagnosis and treatment. Hidden costs arise despite officially free TB treatment and care in China because of doctor recommendations to buy medications to counter side effects of the treatment and the need to visit health care facilities repeatedly. In addition to these costs, and low awareness of treatment options, for migrants, challenges have been reported, as "urban TB control systems tend not to pay enough attention to migrants. They are not required by policies to focus on the needs of migrants and provision of services for them is considered 'extra' work. Many staff have the impression that TB control for migrants is not important" [103]. Unsurprisingly, TB cure rates for migrants in China have consistently been shown to be significantly lower than for residents when they do receive treatment [32,103].

To avoid such barriers in access to HIV/AIDS services when free care is officially available, states and international agencies and donors need to formulate programs to specifically address internal migrants' needs. Crucially, cross-regional linkages need to be developed to facilitate the transition from one regional health authority's care to the next, where health care is not administered at a national level. The process of developing specialized services for internal migrants should include an assessment of the extent to which differences in treatment protocols and drug combinations across regions within a country or across health care providers within the country impede internal migrants' continuity of care. Additional programs facilitating migrants' care could include providing translators who could translate to the languages internal migrants to the region frequently speak, providing mobile outreach services or transport from areas where internal migrants live to health centers, educating health care providers as to migrants' particular needs and rights, or holding patient education sessions geared toward migrants.

## Conclusion

Internal migration is a reality of life for millions of people, and often a pre-condition for the economic and social development on which governments, families, and communities rely. In times of financial crisis, the need to serve and support those people who have been the engine of economic growth is all the more acute. Social protection and health care systems need to keep pace with the reality of internal migration. The criticism of human rights researchers in China, that: " [t]he *hukou *system has always been unfair to migrants, but the economic crisis makes it downright punitive by denying many long-term migrants who have literally built the cities they live in a social welfare net when it is needed most" [16] can be generalized wherever residence-based restrictions on health services are in place. In the face of HIV and other transmissible diseases, serving internal migrants is a public health imperative. Furthermore, it is an obligation that governments have taken upon themselves under international human rights law, including through their commitment to attaining universal access to HIV prevention, treatment, care and support. In national and international efforts at system-wide change in the wake of the economic crisis, taking account of the health needs, human rights, and development goals of internal migrants will be critical to better supporting the next generation of the international economy's workers.

## Summary

Worldwide, far more people migrate within their country than out of it. Internal migrants are those individuals who change residence from one civil division to another within their country of origin. Gaps in internal migrants' access to HIV/AIDS services--either as a result of official restrictions or cultural and linguistic barriers--have significant consequences: individuals are less able to access prevention, care and treatment, states are less able to realize goals of reduced HIV incidence and burden of disease, and the public health community may face the emergence of drug-resistant strains resulting from interruptions in treatment. This article describes some of the barriers to access to HIV/AIDS-related services faced by internal migrants when they move from their place of origin, highlighting three countries--China, Russia, and India--that have strict internal migration restrictions, and linguistic and cultural barriers to HIV/AIDS prevention and treatment. Given that international human rights law guarantees individuals lawfully within a territory the right to free movement within the borders of that state, a right to the highest attainable standard of health care, and the principle of non-discrimination, states have a duty to provide a core minimum of health care services including HIV prevention and treatment to internal migrants on a non-discriminatory basis. Targeted HIV prevention programs and the elimination of restrictive residence-based eligibility criteria are also necessary to ensuring internal migrants' equal rights to HIV prevention and treatment.

## Competing interests

The authors declare that they have no competing interests. This research was supported by Human Rights Watch, an independent, nongovernmental organization.

## Authors' contributions

Both authors wrote, edited, and approved the final manuscript.
